# ‘*He was a brilliant student but became mad like his grandfather’*: an exploratory investigation on the social perception and stigma against individuals living with mental health problems in Bangladesh

**DOI:** 10.1186/s12888-022-04359-3

**Published:** 2022-11-14

**Authors:** Tunvir Ahamed Shohel, Nishad Nasrin, Fariha Farjana, Taufiq-E-Ahmed Shovo, Aisha Rahman Asha, Morsheda Akter Heme, Ashraful Islam, Pranto Paul, Md. Tanvir Hossain

**Affiliations:** 1 Sociology Discipline, Social Science School, Khulna University, Khulna 9208 Khulna, Bangladesh; 2 Economics Discipline, Social Science School, Khulna University, Khulna 9208 Khulna, Bangladesh; 3 English Discipline, Arts and Humanities School, Khulna University, Khulna 9208 Khulna, Bangladesh

**Keywords:** Mental health, Person living with mental health problems, Student, Social stigma, Social perception, Bangladesh

## Abstract

**Background:**

Worldwide, mental health issues constitute a substantial threat to people’s social, economic, and mental well-being and contribute significantly to many fatalities each year. In Bangladesh, people with mental health issues typically delay contacting health professionals because they prefer traditional or religious healers. Moreover, the situation is exacerbated by a lack of awareness, social stigma, and negative perception of sufferers of mental health issues on the part of families and the community. Therefore, this paper investigates the social perception and stigmatization of individuals living with mental health problems and their caregivers in Khulna, Bangladesh.

**Methods:**

Data were collected from university students with concurring mental health issues as well as their closest caregivers, who had in-depth knowledge of the problem and a willingness to take care of the individuals with mental health issues. Following the criteria for data collection, eight individuals living with mental health problems and five caregivers were purposively selected for this research. A semi-structured in-depth interview guide was used for the confidential data collection process, which took place in November and December 2021, and each interview lasted 40–50 min on average.

**Results:**

This study used thematic analysis to present the results; the findings showed that: individuals afflicted with mental health problems sought both medical and spiritual support to recover. Those with mental health issues who received positive family support recovered relatively faster than those who did not. However, negative social perception and stigmatization were the key impediments for individuals suffering from mental health problems and their families, as they found it difficult to discuss their issues with relatives and communities when attempting to access support or seek remedies. Moreover, the commonality of social stigmas, such as labeling mental health problems as equal to ‘madness,’ hindered disclosure to family members, peers, and the community.

**Conclusion and recommendations:**

In Bangladesh, the majority of individuals living with mental health problems are stigmatized and do not receive emotional support. Hence, we suggest nationwide community-based awareness-building programs to promote more positive perceptions of the fight against mental health disorders. Furthermore, counseling and awareness-building programs for effective discouragement of non-scientific remedies such as spiritual healing, as well as diagnosis and medication at the primary stage of sickness, are recommended for early detection and better medical assistance.

## Background

Globally, mental health issues, including anxiety, depression, and bipolar disorder, account for about 15% of deaths [1]. Despite practical steps to combat these prevalent mental health concerns, millions worldwide, particularly women in lower- and middle-income nations, suffer from various mental illnesses [2]. In the wake of the COVID-19 epidemic, mental health problems have escalated to an all-time high, affecting people of all ages and professions, especially in developing nations like Bangladesh; and these disorders include depression, anxiety, stress, sleep issues, and addiction [3–8]. According to a recent nationwide survey, 18.7% of adults in Bangladesh suffer from mental health disorders, with depression and anxiety more prevalent among women and the elderly [9]. During the COVID-19 pandemic, however, the prevalence of depression and anxiety among adults, particularly students, ranged from around 50% to more than 80% [3, 6–8]. Although mental health disorders are on the rise in Bangladesh, they are not perceived as a health concern, are not prioritized for healthcare treatments, and are frequently disregarded [10].

In Bangladesh, people with mental health issues often delay seeking medical help, owing to a lack of awareness and shortage of psychiatrists in general, particularly in rural and sub-urban settings [10]. Moreover, preference for traditional or religious healers further delays actual treatment. However, the most pressing reason for the delay is social stigma – ‘a negative stereotyping and prejudiced beliefs and discriminatory behavior towards individuals living with mental health problems and their families’ – [11]. Social stigma, as the Canadian sociologist Goffman described, is a state that disqualifies individuals from full social acceptance [12]; this disqualification also prevents those living with mental health problems from seeking help, as they are conflicted about the way people might react to or judge their help-seeking behavior [13]. In fact, social stigma on the part of relatives, the wider community, and in some cases, medical personnel deters individuals living with mental health problems from seeking treatment or, in other cases, causes them to stop treatment altogether [14–16]. A study in Indonesia suggested that there are different forms of social stigma, including personal, public, family, and employment, toward professionals and other individuals living with mental health problems [15]. Studies suggest that the labeling of ‘non-normal’ leaves individuals with a ‘feeling of shame’; thus, they avoid social contact and isolate themselves from the community and, to some extent, from family members [14, 15, 17].

In addition to this labeling of individuals living with mental health problems, their family members are also stigmatized and viewed unfavorably by the community [14, 17]. According to a study conducted in Norway, caregivers are frequently discriminated against by family or coworkers for helping individuals living with mental health problems, which put additional strain on their mental health [18]. Moreover, caregivers experience a strained relationship with their care recipients. For example, a study on caregivers in Australia suggested that they often sustain mental health burdens, such as tension and worry, and are often mistreated by their friends or relatives, leading to poor sleep quality [19].

Although many studies have been conducted in countries other than Bangladesh regarding the intensity and consequences of social stigma against individuals living with mental health problems and their caregivers [11, 14, 15, 17–19], to the best of our knowledge, there is no empirical study that traces the social stigma of individuals living with mental health problems and their caregivers in Bangladesh. Hence, this study was designed to explore the experience of these individuals and caregivers and to suggest strategies for combating the societal labeling of people with mental health issues.

## Methodology

### Research design

The prime focus of this study was to investigate the social stigma associated with mental health issues. On this ground, a qualitative approach was used because it would facilitate our understanding of the rational foundations upon which our subjective experiences, judgments, and opinions might build when explaining human behavior, thought, and action [20].

### Study subject

The target group of the research was university students experiencing mental health issues. Caregivers of individuals afflicted with mental health issues were also recruited in order to attain insight into their loved ones’ conditions. Some specific criteria were followed when recruiting the student participants: (i) the participant must be a student, (ii) enrolled in a regular educational program. When recruiting caregivers, the criteria were: (i) they must be close to the individuals living with mental health problems, (ii) have in-depth knowledge and a willingness to take care of the individuals living with mental health problems. Furthermore, we used purposive sampling to select our eight student participants from non-clinical settings, as it enabled us to make initial contact with a small network of individuals who were relevant within the field of investigation and accordingly utilize them as a springboard to expand the scope of the research [21]. When selecting the caregivers, we used convenience sampling because it helped us to gain important insights into any specific investigation issues arising from the minimal number of sample cases [22, 23]. In qualitative research, it is possible to make generalizations even from a very small number of sample participants [21]. Accordingly, it is also worth mentioning that due to the sensitive nature of the research and the confidentiality needs of the participants, the sample size was very limited in number.

### Interview outline

We designed a semi-structured interview schedule in order to gather first-hand information, based on a review of the pertinent literature. The interview schedule was broken down into four parts. Individuals receiving mental health care were initially questioned about their personal and family background and their living arrangements. In addition, questions about the individuals living with mental health problems and caretaker’s mental health experiences and social responses were included in Sects. 3 and 4, respectively.

### Data collection

The study encompassed two stages of data collection. In the first stage, eight individuals living with mental health problems living in Khulna were interviewed over two consecutive months (November and December 2021); in the second stage, five in-depth interviews were conducted with the caregivers of the individuals who were living with mental health problems (who stayed with and took care of the individuals living with mental health problems).

Prior to data collection, informed consent was sought from the participants for their voluntary involvement. All the interviews were conducted in Bangla, and there were no pauses or interruptions. Each interview lasted about 40–50 min on average. With participants’ informed consent, the whole interview was recorded verbatim for later replay. Interviews were held in the strictest confidence, and every participant had the option to withdraw from the study at any time, with no questions asked. As such, we were able to gather data while maintaining a cordial connection with the participants, since we were in an objective position. To ensure the accuracy of the data and eliminate any prejudice, we exercised prudence during the interviews and adhered to the golden rule of unconditional acceptance by attentively listening, asking clarifying questions, and providing comprehensive responses. However, it appeared that the research had become saturated after the sixth interview, and data collection was halted after the eighth interview. In this regard, we must clarify that in qualitative research, saturation is often claimed to be somewhat justified or explained [21]. It is worth mentioning that small sample size, particularly in interview-based qualitative research, helps researchers to increase their chances of gaining participants’ trust and collecting detailed information, which was important for this study [21]. Because individuals living with mental health problems in Bangladesh are often stigmatized, therefore, they do not open to people due to trust issues.

### Data analysis

Following the completion of the interviews, the transcribed data were coded and interpreted into themes using the qualitative data analysis software NVivo 12. Each author contributed to the summarizing of essential points and the setting of the stage for the themes. Data were analyzed using thematic analysis because using thematic analysis, it is possible to see which themes are most essential to the explanation a phenomenon under study. Moreover, the results of a thematic analysis draw attention to the most prominent clusters of meaning within the data set. We established a connection between the study’s findings and external circumstances by correlating the theme’s frequency of occurrence. In addition, following each interview, we deliberated on what to include in our preliminary analysis and reached a consensus. Meanwhile, we carefully examined and evaluated the interview data to resolve any discrepancies and, in extreme circumstances, rule them out completely.

## Results and findings

### Background information of the informants

Of our 13 in-depth interviewees, eight informants were first-hand sufferers of mental health issues who were (and still are) undergoing medication, and the remaining five were caregivers of those individuals living with mental health problems (see Table 1). According to the background data provided by the informants six of the eight individuals living with mental health problems were unmarried male students, and four of these male students had no earnings. The other two male students had an income from private tutoring to other students. In addition to the six male students, there were two married female informants. Most (6) of the informants with mental health issues were living with their family members such as parents or husbands. The background data also showed that all eight individuals living with mental health problems were aged between 21 and 25 years. All informants had first reported their mental health problems in the previous ten years (between 2011 and 2021). Three of the five informants who provided care were housewives, while the other two were men who were involved in business.

**Table 1:** Background information of the informants

Individual living with mental health problems and their Caregivers	Sex	Age	Education	Occupation	First detected
Informant 1	Male	22	BSS (Third Year)	Student & Private tutor	In 2020, when he was a third-year student of bachelor’s degree
Informant 2	Male	22	BA (Fourth Year)	Student & Private tutor	At the end of 2015
Informant 3	Female	24	Degree (Second Year)	Student & Tailor	In the year 2018
Informant 4	Female	24	BSS (Fourth Year)	Student & Housewife	In April, 2020
Informant 5	Male	24	BSS (Third Year)	Student	In 2012, when he was an SSC examinee
Informant 6	Male	23	BSS (Third Year)	Student	In May, 2021
Informant 7	Male	25	BSS (Third Year)	Student	In April, 2020
Informant 8	Male	21	BA (Second Year)	Student	In 2015, when he was an SSC Examinee
Caregiver 1	Female	43	SSC	Housewife	Not applicable
Caregiver 2	Female	48	Class 5	Housewife	Not applicable
Caregiver 3	Male	32	Degree (Complete)	Businessman	Not applicable
Caregiver 4	Male	48	B. Com (Complete)	Business	Not applicable
Caregiver 5	Female	40	Degree (Complete)	Housewife	Not applicable

^Throughout this paper who provided information to this study are addressed as informants and caregivers. Whereas informants refer to the individuals who are living with mental health problems and caregivers are those who stayed with and took care of the individuals living with mental health problems^^*BSS* Bachelor of Social Science, *BA* Bachelor of Arts, *SSC* Secondary School Certificate, *B.Com* Bachelor of Commerce^

Based on the qualitative data we gathered from the field, our results generated four significant themes, including some subsets of themes. These were:

1. Mental health-related problems and seeking remedies.
2. Family perception, support, and response to mental health-related problems.
3. Social stigma and labeling of madness.
4. Reaction from the community to mental health related problems.

#### Mental health-related issues and seeking remedies

The qualitative data shows that six of the informants living with mental health problems had received supportive responses from their families. However, all but one of these informants claimed that their family members were hesitant to talk about these issues with strangers. These families considered mental health problems to be a private family issue. Moreover, they acknowledged the need for confidentiality due to the community’s poor perception of matters relating to mental health. Additionally, the social stigma associated with mental illness, which is linked to insanity, can cause a family to suffer from ongoing humiliation and misery. All of the informants concurred that the community’s unfavorable attitudes and responses would negatively impact individuals living with mental health problems and trying to overcome the situation.

Although the positive response from the family regarding mental health issues was sometimes early and sometimes delayed, it always significantly contributed to improving the individual’s mental health. Two themes illustrate families’ reaction to the informant’s mental health issues:

I. Seeking medical support.
II. Seeking spiritual support/faith healing.

##### Seeking medical support

At least six of the informants living with mental health problems said that their families had sought the support of a clinician (such as a psychiatrist) in order to address the health issue. Informant 1 shared his experience in this regard:

From the very early stages of my problem, I was content with my family’s support. My family immediately contacted a psychiatrist to seek remedies and motivated me to spend more time with my closest friends. They positively believed that spending more time with my peers and family members would aid me in fast overcoming my mental health problems.

Although some (at least three) informants were hesitant to share their mental health issues with their family members independently, the family members observed the anomalies in the individual’s behavior and took positive action to seek treatment. For instance, informant 4 reported:

I have been suffering from mental health problems for more than a year. My medication is ongoing as I still have not recovered properly. Truly speaking, I neither shared nor explained this to my family at the initial stage of my problem. However, my family members, particularly my elder sister, observed my behavioral abnormalities and understood that I was going through some mental disturbances. They immediately sought guidance from a psychiatrist and consulted for remedies.

Informant 5 reported a slightly different but similar experience to informant 4. The experience of informant 5 demonstrates the importance of family support for an individual living with mental health problems which is continuing with long-term treatment. Recovery from mental health issues takes time, therefore ongoing family support is required. As informant 5 said:

I was out of control at the beginning of my mental health problems. I did not understand the process and anomalies I faced then and was annoyed to share this with anyone in particular. Fortunately, my family members could detect my problems immediately, and they took me to a psychiatrist. My family took me to a different psychiatrist because the first one failed to help me much. We found that the second psychiatrist was helpful with the counseling and medication.

##### Seeking spiritual support/faith healing

Outside the medical support system, many families still believe that mental health issues are a spiritual problem and seek spiritual or faith-based solutions. The qualitative data showed that two of the interviewees had a spiritual definition of their mental health problems, and their families had sought faith based healing for the problem. These families had also searched for a religious solution to the issue. However, these two informants’ lower level of education and their location in isolated rural places may be why they chose not to seek emergency medical assistance. In rural areas, mental health difficulties are still considered to have a paranormal explanation, and many people choose to turn to spiritual therapies. The experience of informant 3 demonstrates this:

Upon sharing, my family ignored my mental health issue. They considered my problem as a paranormal issue and contacted a local *Kabiraj*^1^. Within months, they consulted three more local *Kabiraj*. All the *Kabiraj* concluded that my problems are activities of dissatisfied souls; therefore, they applied their incantation and traditional treatments over me. This took months while the situation worsened. After months of the *Kabiraj’s* treatment, my family finally shared my issue with one of our relatives. My relative instantly suggested contacting a psychiatrist.

The findings from the aforementioned two themes suggest that the informants’ families often had mixed perceptions of mental health-related problems. The interviewees indicated that those who received immediate and proper family support and consultation with a psychiatrist had a quicker recovery than those who were taken to local faith healers. Informant 2, who was taken to a local *Kabiraj*, asserted that:

My family members contacted many *Kabiraj* and *Fakir*^2^, and they told us that it was the influence of an evil spirit, and someone might have performed black magic on me. However, after receiving *Kabiraji* treatment, my situation deteriorated. Until my family took me to a medical practitioner (psychiatrist), my condition did not improve.

#### Family perception, support, and response to mental health-related problems

Among our 13 informants, five were direct and close caregivers of the individuals who were living with mental health problems. All five caregivers admitted that they considered their family member’s mental health problem a critical issue that needed to be resolved. However, most of them also reported that they had not anticipated this kind of health crisis in their family. In each case, the whole family had eventually become concerned after learning about the mental health problems of their loved ones. One caregiver (Caregiver 4) explained that:

When I first heard about my daughter’s mental health-related problem, I was puzzled and sad. I must confess that the whole family underwent a mental health crisis hearing my daughter’s case.

All the caregivers reported that their closest who suffers with mental health-related problems had caused the family to undergo much propagation and speculation, hampering their personal, family, and public images and lives. As a result, the original choice of action was not always in the informant’s favor. A mental health issue for one individual appears to be a robust depression and anxiety producer for the family as a whole. Sometimes, negative speculation worsens the situation. For instance, Caregiver 3 explained their experience of this:

First, all my family members were somewhat speculative about my wife’s mental health issues. I must admit that it was not a positive reaction and speculation, that put the family into a disturbance. We wondered if my wife was having an extramarital affair with someone else. She also acted like she had a mental health disorder that was not real. Under this speculation, we sent my wife to her parents’ house.

A family may delay vital medical support due to negative speculation. Community beliefs can lead the family to consult a religious healer or adopt spiritual solutions. These beliefs are sometimes inherited. In this regard, Caregiver 2 reported that:

When we realized that my son was suffering from mental health issues, we took him to the *Kabiraj.* At an early age, his grandfather also had this problem and was taken to the local *Kabiraj and Fakir*. Following the experience, we gradually contacted many *Kabiraj* and *Fakir.* All of them said that an evil spirit had captured my son’s soul. There was also speculation from the *Kabiraj* that someone may have used black magic on my son. We had faith in their stories and continued with their treatments.

However, those families who utilized faith healing or spiritual solutions ended up prolonging the individual’s mental health crisis. All the informants who had contacted *Kabiraj and Fakir* at an early stage for a solution said that they later had to consult specialized medical practitioners in order to ensure a better result for their individuals living with mental health problems.

#### Social stigma and labelling of madness

Our early findings demonstrated how family support is essential when dealing with mental health-related problems. Nevertheless, both individual living with mental health problems s and families also had concerns regarding the acceptance level from the community. Therefore, in determining whether an individual or family will communicate such issues at a mass level, it is crucial to understand how the wider community responds to and accepts mental health difficulties.

The in-depth interviews showed that although four informants did not face any labeling, their community support system was lacking. Although individuals living with mental health problems could have benefitted from community support, they admitted to facing hurdles in the shape of friends, neighbors, and other community members.

The other four informants who were living with mental health problems said they had faced labeling, were stigmatized, and felt unwanted by many of their friends and neighbors. They found that people used the terminology of ‘madness’ as equivalent to mental disorder. All four of these informants asserted that people of all ages (young to old) had laughed at them in their presence or absence. They also found that some people made up irrelevant rumors or engaged in superficial talk such as stating that the informants’ ‘madness’ (equal to a mental disorder) had resulted from their sins or misdeeds. Informant 3 reported this type of upsetting experience:

I was very depressed to see my closest friends’ reactions. Besides my mental disturbance, their behavior also put me further into depression. Their disregard toward my illness was intolerable, and their rumors about me worsened my condition and left me traumatized. For instance, many of them suspected me of having an extramarital affair. They even considered my symptoms of illness to be unreal and thought that I was acting. Moreover, they labeled me as mad or crazy.

Similarly, it was challenging for families to talk about the informant’s mental health problems in public. Damaging community acceptance with ‘madness’ usually risks the respect and dignity of the family. Thus, almost all the caregivers admitted that they did not want to share their family problems with the wider community. They argued that harmful comments from relatives and neighbors would stigmatize the family and negatively impact the informant’s health. Of this experience, Caregiver 4 asserted that:

Though we did not allow my daughter to interact with outsiders, we failed to stop our neighbors from intimidating her. We heard that some of our relatives and neighbors started mocking my daughter in her absence. Their mockery humiliates our daughter and puts our whole family into embarrassment. Unfortunately, we did not get our community’s support; instead, they taunted us verbally and mentally. The people in the community labeled my daughter as mad and us a mad family.

Caregiver 5 also shared her negative experiences:

Despite his mental health issues, I encouraged my son to maintain a good relationship with our relatives and friends. However, my son was labeled as mentally unbalanced and mad. His university friends and cousins started ignoring him. The humiliation in the social support system was very stressful for my son.

Although a small number of people acknowledged receiving some support from those close to them, this compassion could not displace the idea of ‘madness’ with regard to issues relating to mental health. Whether supportive or non-supportive, the community as a whole labeled mental health-related issues as ‘madness’, indicating that the individual living with mental health problems was mentally imbalanced or a lunatic. Caregiver 2 expressed her opinion on and experience of this:

Most of our relatives and neighbors were supportive regarding my son’s mental health-related problem. The fact that they refused to stop calling my son crazy hurt us the most. They sympathized with my son but also taunted him with the ‘lunatic’ expression. For instance, some of them said, ‘Ahhh!! (expression of sadness), he was a brilliant student but became mad like his grandfather.’ These expressions in our presence as well as in our absence in public were stressful.

#### Reaction from the community to mental health related problems

Individuals living with mental health problems can either benefit or face challenges from their friends, neighbors, and other community members. Our in-depth interviews showed that half of the individuals living with mental health problems (4) did not experience any labeling, as their neighbors and community members supported them. In addition, the community’s suggestions and mental supports helped the person living with mental health problems an early recovery. Informant 1, who resided in a student hall, stated that:

I shared my problems with my best friends. They were incredibly supportive and helped me to handle my issues. They inspired me and said positive words that there are some ups and downs in everyone’s life, and I must have patience.

Conversely, the rest of the informants (4) experienced labeling stigmatization by friends and family. Moreover, they asserted rigorously that people from all backgrounds and ages had humiliated them and their families. They were labeled as mad and treated as insignificant. Of this experience, informant 3 stated that:

I was traumatized by the intense mental pressure from our close family members. Their suspicion of me and my life was disturbing. As a result of their doubts and rumors, my in-laws started to dislike me, and I was shamed in front of my parents. None of this helped me to improve my mental condition.

## Discussion

This qualitative study aimed to investigate the stigmatization experienced by individuals living with mental health problems and their family members. The results and findings section presented the perspectives of eight individuals living with mental health problems and five caregivers who were interviewed for this purpose, with their experiences related in thematic order. The findings revealed how mental health-related issues and remedies were handled by individuals living with mental health problems and their families. It also showed how family support was provided, considering both the community response and perception of mental health issues.

All informants (individuals living with mental health problem) in this study were between the ages of 21 and 25, with their first experience of mental health difficulties occurring in the previous ten years [24]. This is considered the peak time for the onset of mental illness, which comprises anxiety and depression [25]. In certain instances, family members proactively consulted doctors for medical assistance and permitted the informant to spend more time with their peer groups [26]. However, the individuals living with mental health problems occasionally experienced the reverse. Instead of sharing mental health issues with doctors, some families preferred to keep them confidential. They believed that telling others could violate their family’s code of honor. Carr and Ashby [27] found that people view the identification of mental health-centered issues, such as anxiety and depression, as derogatory and discriminatory. Furthermore, there was a mixed response from family members toward individuals suffering from depression or anxiety. Skundberg-Kletthagen, Wangensteen [18] state that in many cases, relatives have feelings of anger and irritation toward the depressed member of their family, since the situation is restricting them from leading the peaceful life they want to live. For instance, Ahlström, Skärsäter [28] have shown that when a family has a mentally troubled child, it affects the relationship between the spouses, which is sometimes characterized by verbal aggression and accusation. Caqueo-Urízar and Gutiérrez-Maldonado [29] further opine that informants’ family members suffer psychologically and socially due to having a mentally unhealthy person in their household.

To overcome the problems of depression, anxiety, and mental health-centric distress, we identified two genera of support, namely: i) medical assistance and ii) spiritual assistance. Seeking medical assistance was very common among the informants. The pattern of the help-seeking behavior of depressed individuals and their families was context-specific. It was clear that some participants would have preferred to consult a medical specialist or MBBS doctor, but due to financial insolvency and geographical inaccessibility, they instead consulted local village doctors and faith healers [30]. The literature has also found that seeking spiritual assistance is a prevalent practice. Ngoma, Prince [31] report that many individuals living with mental health problems consult traditional healers for spiritual treatment. Many families of mentally distressed individuals strongly believe that depression and anxiety are not a disease but instead a curse resulting from witchcraft and evil spirits [32, 33]. We also found a prevalence of delayed response in terms of treating depressed individuals with proper medication. many of the families strongly believed in the supernatural and magical aspects of causing and curing mental illness [34]. In treating depressed individuals, these family members preferred to consult traditional healers. In developing countries, traditional healers are often seen as the primary agents for treating individuals struggling with psychosocial noise [35]. In this paper, we detected that the culture of preference for traditional healers, i.e., *Fakir or Kabira*, still persists in Bangladesh; Picco, Abdin [36] have also mentioned that adopting poorer treatment can be a mitigation tool and that delays in appropriate treatment have negative consequences for individual’ mental health outcomes.

When a family member first learns that their relative has a mental health issue, their perspective is one of confusion and anxiety. They have no idea what to do and what not to do. Whom should they contact in order to overcome the problem. All of these factors affect their own mental health. This stressful ambience inside the household also makes the depressed individuals more depressed. Breland-Noble, Wong [37] have also detected that individuals struggling with depression are more exposed to family arguments than non-depressed individuals. These arguments relapse the somatic symptoms of depressed individuals and push them into the vicious circle of depression [38]. In addition, sometimes family members become speculative regarding the depressive symptoms of the individual. In this study, we found that one of the depressed female individuals had in-laws who suspected that she was only faking madness and that she might be having an extramarital affair. The findings of Selim [30] are consistent with our result; she argues that people hardly recognize depression, while the participants she surveyed termed it as ‘*China rog*,’ caused by financial weakness. However, Sanz and García-Vera [39] state that an individual can experience depression despite everything else in their life going well.

From the in-depth interviews, our findings show that some informants do not experience the labeling of madness. However, support from their peer groups and surroundings is inadequate. Lauber and Rössler [34] have found that in Asian countries, the tendency of communities to stigmatize and discriminate against individuals living with mental health problems is widespread. Mentally distressed individuals are considered dangerous, which yields an inclination to maintain a safe distance from individuals suffering from anxiety and depression. Therefore, the adverse societal reactions of communities towards mentally unwell individuals are pervasive. This worsens the person living with mental health problems’ condition and limits the scope of their social integration [40]. It also relapses the self-stigma of the mentally distressed individuals which is assimilated with the social stereotypes and causes loss of self-esteem and deterioration of self-efficacy. This ultimately makes the individual and their family members hesitant to socialize. Moreover, many participants reported not knowing that something was wrong — sometimes for years. One participant did not seek treatment because they “didn’t know anything was wrong with me”. A similar situation has been observed among person living with mental health problems suffering from depression in the USA [41].

### Limitations

The selection process of the study location and informants does not provide a full representation, as the study location only represents a single locality among the whole of Bangladesh and the informant groups were a tiny portion of a larger population. Therefore, the findings we have concluded from the data have limitations concerning generalizability to the wider group of individuals living with mental health problems in Bangladesh. Another limitation of this research was that we gathered information directly from the informants living with mental health problems and caregivers, who were susceptive of the issues. However, this rapport building was influential in ensuring a comfort zone between the informants and the interviewers. Other restrictions we must address include problems with funding (as this research did not have any funding), a lack of time, and communication difficulties. For example, in investigating the sensitive topic of mental health problems, we found it challenging to arrange appointments and schedule interviews with individuals living with mental health problems and their caregivers. Communication was another problem, as most informants were hesitant to provide data on this sensitive topic. Moreover, the data were collected during the COVID-19 lockdown; the related safety precautions interrupted the data collection procedures and communication, which posed another challenge for this study. We also acknowledge that further data from a wider range of informants who are living with mental health problems around the nation would be necessary in order to declare the findings generally applicable. However, we believe that apart from these limitations, this current study can potentially contribute to mental health literature and could also be significant for comparing or contrasting data in the relevant field.

## Conclusion and recommendations

The social stigma regarding individuals’ mental health problems hinders disclosure of the problem, especially among the family of individuals living with mental health problems, community, relatives, and peer groups. Both individuals living with mental health problems and their families frequently attempt to keep such problems hidden from the public and their neighborhoods. Usually, society labels mental health problems as ‘madness’ and imposes a social stigma, which causes non-disclosure of the problem on the part of the informant and their family. The community’s perspective on this matter constitutes a restriction on the openness of society, which needs to be addressed delicately. In Bangladesh, as in many other underdeveloped nations, mentally ill people are not adequately understood by the community. Due to the lack of social acceptance, families frequently struggle with regard to telling the wider community about any mental health concerns that affect their family members.

In response to mental health problems, the individuals living with mental health problems and their family members primarily try to find ways to move away from the illness. Therefore, they seek help from psychiatrists. The success of psychiatrists varies; sometimes they are effective and sometimes not. However, a quick response and effort from the mentally unwell person and their family members to improve the mental health condition and regain the mental stability of the individual, as well as a diagnosis, are essential at the initial fast-moving stage of the mental health problem. In this regard, the role of family members, relatives, and peers is essential.

In most cases, a diagnosis and medication at the primary stage of the mental health issue can provide better health outcomes. Therefore, a prompt and visible response to the problem by family members is highly beneficial in combating mental health issues. As such, awareness-building programs using electronic media such as television, radio, and social media could be an effective instrument to upgrade the social lens toward handling and accepting individuals living with mental health problems. Additionally, different awareness programs, as well as government and non-government organizations, should promote medication and pragmatic rehabilitation programs for mentally ill people.

It has been found that when dealing with mental health problems, people frequently seek non-scientifically validated spiritual remedies and local faith healers. This preference occurs due to belief in superstitions and ignorance, which often worsen the individual’s condition. However, counseling and awareness-building programs can effectively prevent use of spiritual or faith-based treatment. Discussion of mental health problems and the procedure of dealing with individuals living with mental health problems by their families, communities, peers, relatives, and societies in the textbook at different stages of education can bring long-term constructive changes in the social settings.

It was also found that one family member’s living history of mental illness can cause the mental health problem of other family members, especially regarding depression, anxiety, and stress, to worsen. Families also often feel that society is looking down on them, which creates an extra mental burden that may prolong their efforts to find a road to recovery for individuals living with mental health problems. Consequently, positive mental support from society, community, peers, and relatives are highly recommended in order to break social perception bottlenecks and boost the acceptance of individuals living with mental health problems.

## Data Availability

All data generated or analyzed during this study are included in this published article.

## Abbreviations

BA: Bachelor of Arts
B.Com: Bachelor of Commerce
BSS: Bachelor of Social Science
Covid-19: Coronavirus disease 2019
MBBS: Bachelor of Medicine, Bachelor of Surgery
SSC: Secondary School Certificate
USA: United States of America

## Footnotes

1 Local/traditional healers who largely expand their practices on faith healing. They are recognized as quacks in the modern health system.

2 Spiritual healers hold strong religious beliefs about treating patients.
